# A Case of Thoracentesis

**Published:** 1875-02-15

**Authors:** Douglas Morton


### A CASE OF THORACENTESIS.

By Douglas Morton, M.D.

*From the American Practitioner, February, 1875.*

A YOUNG mulatto woman was admitted into the City Hospital, August 2, 1874, with the following history gathered from her former physician: four months before she had been delivered, after an easy labor, of a six months' fœtus. She did well for twelve days, when she had a chill, followed by a sharp attack of pleuro-pneumonia of the left side, which after lasting for a fortnight, seemed gradually to pass away. Some days subsequently rigors, as many as two or three daily, occurred, and on examination, a large pleuritic accumulation was discovered in the left side. When admitted to hospital, she was much emaciated, and had rigors, hectic, and night-sweats; a morning temperature of 100.5°, with a rise of one degree in the evening; pulse 120. Dr. Chandler, one of the resident physicians of the hospital, detected a large accumulation of fluid in the left pleural cavity. On August 4th, I drew off by the aspirator about forty ounces of very fetid pus, and for want of something better, introduced a No. 8 female catheter to secure subsequent drainage. No improvement occurred in the symptoms —indeed, the next evening, up to which time a large quantity of pus had escaped through the catheter, I found the patient decidedly worse. At the suggestion of Dr. Chandler, injections of a weak solution of carbolic acid were now made into the cavity daily; and under these, conjoined with brandy, quinine, and nourishing food, the patient's general condition slowly improved. The amount of pus discharged continued considerable; but neither the drainage nor the means for washing out the cavity being altogether satisfactory, I attempted to cut out a section of rib. The patient bore chloroform so badly, however, I had to content myself by simply enlarging the opening with the bistoury. The incision was made between the eighth and ninth ribs, an inch and a half behind the first opening, and through it a tube a quarter of an inch in diameter was carried into the cavity, thus securing complete drainage and making injections easy. I now directed that after the daily washing of the cavity with the carbolic acid solution, there should be injected six grains of quinine suspended in water, and this allowed to remain. I did this with the view of testing the supposed antipyogenic property of quinine,* and to avail myself of its known power over putrefaction of nitrogenous matter by, it is believed, destroying certain organisms which seem to bear some relation to the process.

From this time the patient made rapid improvement, and soon a perfect recovery. November 25th, she told me that within three months after the last operation, she weighed one hundred and thirty pounds, which was, I think, nearly double her weight just at that time. I was unable to make as thorough physical examination of her chest as I wished, but thought the lung had nearly, if not quite, expanded to its normal volume. The drainage-tube had been removed about a month, the discharge of pus having ceased some time before.

The history of this case throughout is, I think, of more than ordinary interest. In the first place, while we have nothing to show for the occurrence of metritis, of uterine phlebitis, or of peritonitis during the first twelve days after the miscarriage—which indeed is an exceptionally long time to intervene before the development of disease incident to the puerperal state—with the broader views we now have of septicæmia, of "embolhæmia," and their relations with puerperal fever, we are bound, I think, to admit a rather strong probability of an embolic origin for the pulmonary and pleural lesions in the case. This probability is strengthened by the fact that the patient's surroundings highly favored the occurrence of such an event, and still more by a circumstance connected with the treatment that I have not mentioned. A small quantity of either the quinine or carbolic mixture might be thrown in without producing irritation, but when a certain limit was exceeded, most violent paroxysms of coughing were excited, during which she expectorated purulent matter. She told me too, that occasionally she could taste the injected drugs. These things, I think, clearly proved a connection between the pleural cavity and some of the bronchial tubes formed by the rupture of an abscess.

There were other features that had important bearing upon the prognosis, and should be mentioned. The right lung was healthy and fairly competent for all the oxygenation required in the reduced condition of the patient; and there was no abdominal complication, tubercular or otherwise, to interfere with nutrition.

But the point in this history of crowning interest to myself, is the rapid improvement that followed immediately upon the topical use of quinine, and this notwithstanding the last tube inserted slipped out a day or two after it was put in, leaving the first to do the whole work; a fact I attach not a little importance to, as it seems to be the present opinion of surgeons that either two openings, or one made very large by removal of a section of rib, are almost essential to a good result. This circumstance not only indicates the value of the quinine treatment, but shows that a case of empyema very unfavorable as to prognosis may get well without having to endure the painful operation (for these are unsafe cases for chloroform) of rib-trephining.

* This is one of a series of experiments which I reported in the *Practitioner* for November, 1874.

---

CROTON CHLORAL.—This agent is the aldehyde of crotonic acid, whilst ordinary chloral is the aldehyde of acetic acid. Croton chloral rapidly produces sleep like the other chloral; but, according to Liebreich, it never slackens the pulse or respiration. It never irritates the stomach, and is looked upon as the best remedy for facial neuralgia. The same author gives croton chloral in preference to chloral where heart disease exists, and in neuralgia of the fifth pair. When ordinary chloral does not induce sleep, it should be combined with croton chloral.—*Lancet*, Jan., 1875.

